# RETRACTION: Downregulation of CRABP2 Inhibit the Tumorigenesis of Hepatocellular Carcinoma in Vivo and in Vitro

**DOI:** 10.1155/bmri/9802538

**Published:** 2026-01-22

**Authors:** BioMed Research International

RETRACTION: Q. Chen, L. Tan, Z. Jin, Y. Liu, and Z. Zhang, “Downregulation of CRABP2 Inhibit the Tumorigenesis of Hepatocellular Carcinoma In Vivo and In Vitro,” *BioMed Research International*, (2020): 3098327, https://doi.org/10.1155/2020/3098327.

The above article, published online on 25 June 2020 in Wiley Online Library (http://wileyonlinelibrary.com), has been retracted by agreement between the handling Editorial Board Member, Goutam Ghosh Choudhury, and John Wiley & Sons Ltd. The retraction has been agreed due to multiple concerns identified with the figures, also raised on PubPeer [1]. On further investigation, additional concerns were noted:

• In Figure 1a, the Normal, Stage I and Stage IV panels overlap with panels A, B and E from Figure 1 of [2], when rotated 180 degrees.
• In Figure 1a, the Stage II and III panels appear similar to panels C and D from Figure 1 of [2], when rotated 180 degrees and horizontally flipped.
• Figure 2a is identical to Figure 6 from [3] and Figure 2a from [4]
• In Figures 3c and 3d:
  o Multiple/all panels are the same as ones shown in Figure 2b (HT29 and SW480) of [5]
  o Multiple/all panels are the same as ones shown in Figure 2b (U87 and LN‐229) of [6]
  o Multiple/all panels are the same as ones shown in Figure 3e (MDA‐MB‐231 and BT‐549) of [7]
  o Bottom right panel of Figure 3c is similar to the bottom left panel of Figure 2d in [8]
• In Figure 3e, multiple panels are the same as ones in Figure 3 of [5] and Figure 4 of [9]
• In Figure 3f, multiple panels are the same as ones in Figure 5 of [5] and Figure 4 of [9]
• In Figure 4a:
  o The HepG2/Bcl‐2 blots when vertically flipped appear similar to the p‐GSK3B blots in Figure 5 g of [10]
  o The Huh7/Bcl‐2 blots appear similar to the LN‐229/c‐Myc blots in Figure 4a of [6]
  o The HepG2/VEGF blots appear the same as the LN‐229/B/Catenin blots in Figure 4a of [6]
  o The HepG2/p‐VEGFR2 blots appear the same as the 5‐HT2cR blots in Figure 4b of [11]
• Figure 5a appears to be identical to Figure 2a of [12]
• In Figure 5c, the K67 images appear to be the same as the control, TCM and DDP images in Figure 1e of [13]

The editiorial board have assessed the concerns, and deemed the results and conclusions unreliable, and therefore the article is retracted.

The authors did not respond to the retraction.
